# Effects of pharmacist intervention on polypharmacy in patients with type 2 diabetes in Japan

**DOI:** 10.1186/s13104-020-05032-2

**Published:** 2020-03-30

**Authors:** Takeshi Horii, Koichiro Atsuda

**Affiliations:** 1 Pharmacy Practice and Science I, Research and Education Center for Clinical Pharmacy, Kitasato University School of Pharmacy, 1-15-1 Kitasato, Minami Ward, Sagamihara, Kanagawa 252-0375 Japan; 2 Shimokitazawa Hospital, 2-8-16 Kitazawa, Setagaya Ward, Tokyo, 155-0031 Japan

**Keywords:** Polypharmacy, Type 2 diabetes, Pharmacy service

## Abstract

**Objective:**

Investigation of polypharmacy in patients with type 2 diabetes revealed that medications administered according to the patient’s symptoms and complaints strongly contributed to polypharmacy. We explored the effects of clinical ward pharmacy service, which evaluated the need for symptomatic treatment, therefore minimizing polypharmacy by reducing inappropriate medications.

**Results:**

The number of drugs (hospitalization vs. discharge: 9 [1–17] vs. 7 [1–16], P < 0.001) and rate of polypharmacy (hospitalization vs. discharge: 75.4% vs. 61.1%, P < 0.001) were significantly lower at discharge. Since hospital admission, the number of drugs increased (n = 6, 11%), remained unchanged (n = 15, 28%), decreased by 1 drug (n = 4, 8%), decreased by 2 drugs (n = 3, 6%), and decreased by more than 2 drugs (n = 25, 47%). Daily drug costs were significantly reduced (hospitalization vs. discharge: $8.3 vs. $6.1, P < 0.001).

## Introduction

In Japan, the number of patients with type 2 diabetes (T2DM) is steadily increasing [1]. Medication used for the management of diabetes and its complications (such as hyperglycemia, microvascular complications, pain, insomnia, and other symptoms) may improve target outcome but can also have side effects that lead to the addition of unnecessary medications to the treatment regimen [2]. We identified that this strongly contributes to polypharmacy (PP) in these patients, resulting in an increased risk of adverse drug reactions, drug interactions, and medication non‐adherence [3–9]. Symptomatic treatment depends on the patient’s symptoms and complaints, such as pain and insomnia, and strongly affects PP [10]. Although there is an urgent need to prevent PP, none of the devised methods have been put into practice at many facilities.

After a patient’s admission at the hospital, a clinical ward pharmacist evaluates inappropriate medications using a pharmaceutical approach and checks for PP. The clinical ward pharmacy service is effective in eliminating PP, as reported in the United States of America and in Europe [11–14]. But so far, no similar studies have been carried out in Japan. Therefore, we investigated how the clinical ward pharmacy service affects the rate of PP in T2DM in Japan.

## Main text

### Methods

An uncontrolled before-after study was conducted at the Shimokitazawa Hospital. The Shimokitazawa Hospital has outpatient and inpatient services, and specializes in the treatment of diabetes. In the hospital, certain wards are prepared to receive patients presenting with acute diabetes-related complications. The main responsibilities of the pharmacy service consist of controlling and dispensing medication, clinical data review, patient counseling, communication with healthcare professionals, and participation in ward rounds. We included patients who were newly admitted to the hospital in November 2017. The exclusion criteria were as follows: (1) type 1 diabetes, (2) age < 18 years, and (3) clinical ward pharmacy service not implemented. We reviewed medical records to retrieve the patients’ clinical information. Since PP is defined as taking six or more drugs, our primary outcome was the reduction in these medications. The secondary outcome was the change in daily drug costs from time of hospitalization to discharge.

Normally distributed numerical data is presented as mean ± standard deviation. Categorical variables were analyzed using Fisher’s exact test and Chi square test and are expressed as absolute numbers or percentages. Differences were regarded as significant when P < 0.05. All statistical analyses were performed using the Stata software (version 10; Stata Corp, College Station, TX, USA). In this study, 1 dollar ($) was considered as equivalent to 100 yen. This study was conducted in accordance with the ethical guidelines for medical and health research involving human subjects. The ethics board of the Kitasato University approved the study (Control number: 17078) and provided permission to review patient records and use the corresponding data.

### Results

Table 1 shows the characteristics of 53 patients who met the selection criteria.

**Table 1:** Patient characteristics

	Mean ± S.D. or median (min–max) or n (%)
	Overall n = 53
Male n (%)	27 (50.9)
Age (years)	68.8 ± 14.0
HbA1c (%)	7.1 ± 1.6
BMI (kg/m^2^)	22.2 ± 4.5
eGFR (mL/min/1.73 m^2^)	71.9 ± 64.1
Diabetes duration (years)	7.9 ± 5.9
Number of drugs	9 (1–17)
Polypharmacy n (%)	40 (75.4)
Hospitalization days (days)	17.2 ± 2.5

Polypharmacy was defined as taking six or more drugs

*HbA1c* hemoglobin A1c, *BMI* body mass index, *eGFR* estimated glomerular filtration rate

**Fig. 1:**
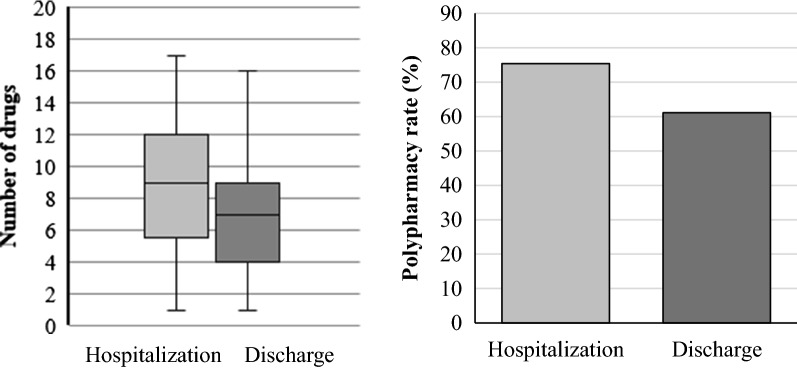
Changes in number of drugs and polypharmacy rate The number of drugs (hospitalization vs. discharge: 9 [1–17] vs. 7 [1–16], P < 0.001) and PP rate (hospitalization vs. discharge: 75.4% vs. 61.1%, P < 0.001) were significantly lower at discharge (Wilcoxon signed rank test)

By comparing the number of drugs and PP rate at the time of hospitalization and discharge, it was possible to observe that both the number of drugs (hospitalization vs. discharge: 9 [1–17] vs. 7 [1–16], P < 0.001) and PP rate (hospitalization vs. discharge: 75.4% vs. 61.1%, P < 0.001) were significantly lower at discharge (Fig. 1).

**Fig. 2:**
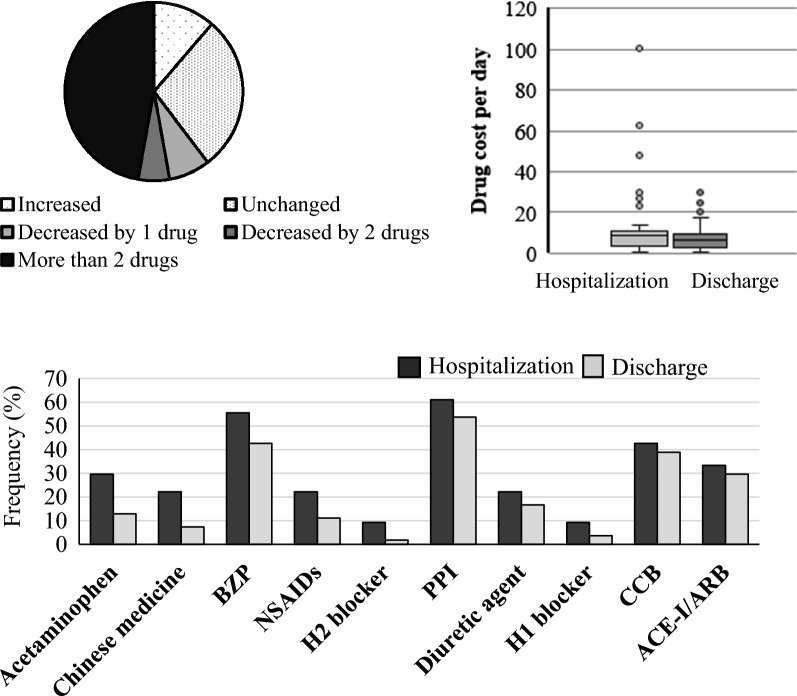
Changes in the number of drugs and daily drug costs and top 10 drugs whose frequency of use changed significantly before and after hospitalization. Changes in the number of drugs since admission were increased (6 patients, 11%), unchanged (n = 15, 28%), decreased by 1 drug (n = 4, 8%), decreased by 2 drugs (n = 3, 6%), decreased by more than 2 drugs (n = 25, 47%). Daily drug costs were significantly reduced when comparing hospital admission and discharge (hospitalization vs. discharge: 8.3 vs. 6.1, P < 0.001, 1$ = 100 yen, Wilcoxon signed rank test). *ACE-I* Angiotensin-converting-enzyme inhibitor, *ARB* Angiotensin II receptor blocker, *BZP* Benzodiazepine, *CCB* Calcium channel blocker, *NSAIDs* Non-Steroidal Anti-Inflammatory Drugs, *PPI* Proton pump inhibitor

Since hospital admission, the number of drugs increased (n = 6, 11%), remained unchanged (n = 15, 28%), decreased by 1 drug (n = 4, 8%), decreased by 2 drugs (n = 3, 6%), and decreased by more than 2 drugs (n = 25, 47%). Daily drug costs were significantly reduced upon comparing cost on the day of hospital admission with that on the day of discharge (hospitalization vs. discharge: $8.3 vs. $6.1, P < 0.001). We have also analyzed which drugs registered the most pronounced reduction in terms of frequency of use. In this aspect, acetaminophen was the drug whose frequency of use dropped the most, followed by angiotensin-converting-enzyme inhibitor and angiotensin II receptor blocker (Fig. 2).

### Discussion

In our study, we determined factors that strongly affect PP through multivariate analysis. The results of this study suggest that by using the existing clinical ward pharmacy service, it is possible to lower the rate of PP and total drug costs per day, without a significant additional monetary investment in the care of patients.

In Japan, medical expenses are increasing not only for older adults, but also for diabetes patients. According to the statistics of the fiscal year 2019, Medical expenses are over $12 billion in Japan [15]. Therefore, there is an urgent need to reduce medical costs, possibly by avoiding unnecessary medication and by reducing PP. However, in chronic diseases characterized by hyperglycemia and hypertension, it can take up to a year to determine the therapeutic effects of the medication, making it difficult to decide if the drugs should be discontinued during the patient’s stay at the hospital. In contrast, the effects of drugs used for symptomatic treatment are fast-acting, allowing one to evaluate the effects of drug discontinuation in the short term. Medication used for symptomatic treatment is strongly responsible for PP in patients with T2DM and there are previous reports which show that drugs which have similar functions are frequently stopped [16]. Thus, we believe that active intervention in symptomatic treatment can be effective in addressing PP. Although we only observed a reduction in daily drug costs of about $1.7, the reduction of PP per se helped improve the quality of life (QOL) by reducing the treatment burden.

Beers criteria and STPP criteria are used as a screening tool for potentially inappropriate medications (PIMs) in PP [17, 18]. In Japan, the “Guidelines for medical treatment and its safety in the elderly” were proposed in 2005 by the Japan Geriatrics Society [19]. As these are screening tools for the older adults, they should not [10]. In our study, we extracted risk factors using multivariate analysis, which could prove useful for addressing PP in T2DM patients under 65 years of age.

Efforts to eliminate PPs include the use of screening tools such as the STOPP criteria and the establishment of specialized outpatient clinics with a multidisciplinary team consisting of doctors, nurses, and pharmacists focusing on the reduction of PP [20–24]. In a study conducted in Japan that extracted PIMs using the STOPP criteria and intervention of pharmacists, it was reported that 28.0% of the drugs that corresponded to STOPP criteria were changed [22]. On the other hand, all drugs changed in this study were done so according to the indication of pharmacists. For this reason, we do not recommend a change in treatment based exclusively on fixed criteria; symptoms differ between patients, and, therefore, the decision to discontinue a drug should be done on a case-by-case basis. In addition, while criteria and efforts to introduce new medical resources are effective in eliminating PP, at present, many facilities cannot enforce them. In fact, the New Health Care Fee, which is included among these new measures to stop PPs, covers only 16.5% of all hospitals [25]. Therefore, PP-reducing methods that take advantage of already-existing services and do not require any further resources should be considered. In our study, pharmacist interventions for PP were conducted for all ages. As a result, the PP rate was significantly reduced, and 53% of patients met the calculation requirement for the The New Health Care Fee by having reduced two or more PIMs.

### Conclusions

We believe that the results of this study will foster further efforts in reducing PP in T2DM in Japan and Asia. By eliminating PP, medical costs are reduced, and the number of drugs taken is reduced, which increases patient satisfaction and contributes to improved treatment adherence. As a result, it is speculated that a reduction in PP has an impact on clinical outcomes such as QOL and Hemoglobin A1c levels. Based on the promising results of this pilot study, we are planning to carry out a larger intervention study in the future.

## Limitations

There are several limitations in this study. First, we did not compare this study with cases where no intervention was performed, and we think it is necessary to examine the details of the effectiveness of this approach in the future. Finally, since interventions are centered on drugs for symptomatic treatment, there is still room for intervention in PIMs.

## Data Availability

The datasets used and/or analyzed during the study are available from the corresponding author on reasonable request.

## Abbreviations

ACE-I: Angiotensin-converting-enzyme inhibitor
ARB: Angiotensin II receptor blocker
PIMs: Potentially inappropriate medications
PP: Polypharmacy
QOL: Quality of life
T2DM: Type 2 diabetes
